# The evolution of *Dscam *genes across the arthropods

**DOI:** 10.1186/1471-2148-12-53

**Published:** 2012-04-13

**Authors:** Sophie AO Armitage, Rebecca Y Freiburg, Joachim Kurtz, Ignacio G Bravo

**Affiliations:** 1 Institute for Evolution and Biodiversity, University of Münster, Hüfferstrasse 1, 48149 Münster, Germany; 2 Unit of Infections and Cancer, Catalan Institute of Oncology (ICO), Gran Via de L' Hospitalet, 199, 08907 L'Hospitalet de Llobregat, Barcelona, Spain

**Keywords:** Alternative splicing, *Dscam-like*, Gene duplication, Ortholog, Co-ortholog, Paralog

## Abstract

**Background:**

One way of creating phenotypic diversity is through alternative splicing of precursor mRNAs. A gene that has evolved a hypervariable form is *Down syndrome cell adhesion molecule *(*Dscam-hv*), which in *Drosophila melanogaster *can produce thousands of isoforms via mutually exclusive alternative splicing. The extracellular region of this protein is encoded by three variable exon clusters, each containing multiple exon variants. The protein is vital for neuronal wiring where the extreme variability at the somatic level is required for axonal guidance, and it plays a role in immunity where the variability has been hypothesised to relate to recognition of different antigens. *Dscam-hv *has been found across the Pancrustacea. Additionally, three paralogous non-hypervariable *Dscam-like *genes have also been described for *D. melanogaster*. Here we took a bioinformatics approach, building profile Hidden Markov Models to search across species for putative orthologs to the *Dscam *genes and for hypervariable alternatively spliced exons, and inferring the phylogenetic relationships among them. Our aims were to examine whether *Dscam *orthologs exist outside the Bilateria, whether the origin of *Dscam-hv *could lie outside the Pancrustacea, when the *Dscam-like *orthologs arose, how many alternatively spliced exons of each exon cluster were present in the most common recent ancestor, and how these clusters evolved.

**Results:**

Our results suggest that the origin of *Dscam *genes may lie after the split between the Cnidaria and the Bilateria and supports the hypothesis that *Dscam-hv *originated in the common ancestor of the Pancrustacea. Our phylogeny of *Dscam *gene family members shows six well-supported clades: five containing *Dscam-like *genes and one containing all the *Dscam-hv *genes, a seventh clade contains arachnid putative *Dscam *genes. Furthermore, the exon clusters appear to have experienced different evolutionary histories.

**Conclusions:**

*Dscam *genes have undergone independent duplication events in the insects and in an arachnid genome, which adds to the more well-known tandem duplications that have taken place within *Dscam-hv *genes. Therefore, two forms of gene expansion seem to be active within this gene family. The evolutionary history of this dynamic gene family will be further unfolded as genomes of species from more disparate groups become available.

## Background

**Figure 1:**
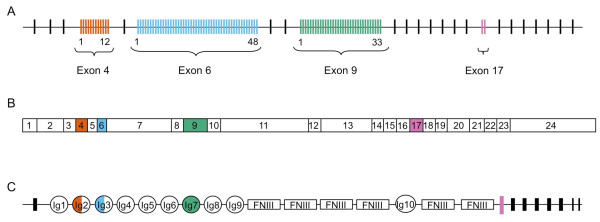
**(A) *Dscam-hv *genomic DNA for *Drosophila melanogaster***. The gene consists of 20 constant exons (shown as black lines), mutually exclusive alternative splicing occurs for exons 4 (red lines), 6 (blue lines), 9 (green lines) and 17 (purple lines); one of 12 exon 4 alternatives, one of 48 exon 6 alternatives, one of 33 exon 9 alternatives and one of two exon 17 alternatives are present in each mRNA. This enables the vast number of 12 × 48 × 33 × 2 = 38,016 potential splice variants. **(B) *Dscam-hv *mRNA**. Constant exons are shown as white boxes. Exons that undergo mutually exclusive alternative splicing follow the same colour scheme as for the genomic structure. Endodomain exons 19 and 23 can be contained or lacking [8], which increases the number of potential isoforms to 4 × 38,016 = 152,064. **(C) Dscam-hv protein structure for *D. melanogaster***. The alternatively spliced exons encode the N-terminal half of Ig2 (exon 4 in *Drosophila*); the N-terminal half of Ig3 (exon 6 in *Drosophila*), all of Ig7 (exon 9 in *Drosophila*), and the transmembrane domain (Exon 17 in *Drosophila *(figure after [6]).

The biological complexity of an organism does not appear to correlate with the number of protein-coding genes in that organism. This is exemplified by comparing the human with the nematode *Caenorhabditis elegans *genome, where roughly similar numbers of genes (20,000-25,000 and 19,000, respectively) result in organisms with startlingly contrasting complexity [1,2]. A pervasive contributor that may in part account for this is alternative splicing of precursor messenger RNA, creating transcriptomic and proteomic diversity [3-5]. A particularly remarkable example is the *Down syndrome cell adhesion molecule *(*Dscam*) gene in *Drosophila melanogaster*, which can potentially generate 38,016 mRNA isoforms via mutually exclusive alternative splicing of exons that encode the ecto- and transmembrane domains [6]; this number increases to 152,064 when one considers independent alternative splicing of exons within the endodomain ([7,8]; Figure 1). The latter figure accounts for more than eleven times the number of genes in *D. melanogaster*'s genome [9]. Mutually exclusive alternative splicing occurs for ectodomain encoding exons 4, 6 and 9 and transmembrane exon 17 ([6]; Figure 1A). One of twelve exon 4 alternatives, one of 48 exon 6 alternatives, one of 33 exon 9 alternatives and one of two exon 17 alternatives are present in each mRNA ([6]; Figure 1B), and endodomain exons 19 and 23 can be contained or lacking [8]. Because of the extreme number of isoforms possible, *Dscam *has more recently been referred to as hypervariable, *Dscam-hv *[10], a nomenclature that we also adopt in this paper, but it is noteworthy that earlier papers do not use the -*hv *suffix (*e.g*. [6]).

The *Dscam-hv *gene is a member of the Immunoglobulin (Ig) superfamily [11]. In *D. melanogaster *it plays an essential role in neuronal wiring: it is an axon guidance receptor [6] and it ensures that olfactory receptor neurons synapse in the correct target [12]. The importance of isoform diversity in neuronal wiring and self-recognition has since been uncovered [13-16]. In 2005 Watson and co-workers [17] discovered that depletion of *Dscam-hv *impairs the ability of *D. melanogaster *haemocytes to phagocytose bacteria, spurring research into the immunological role that it could play in *Drosophila *and other pancrustaceans (i.e., the clade consisting of crustaceans and insects) [17-20]. It has been hypothesised that the large diversity of *Dscam-hv *isoforms could provide specificity for antigen recognition [21-23], however evidence to support this hypothesis is limited. *Dscam-hv *is particularly interesting because of its versatility due to the extreme diversity that it encodes, and also because the nervous and immune systems may exert different selection pressures on this gene [24].

**Figure 2:**
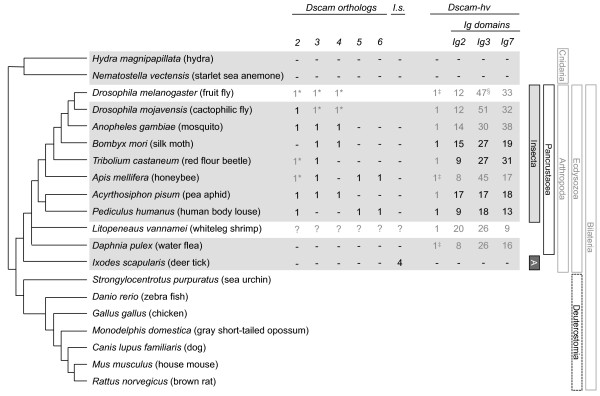
**Phylogenetic relationships between the species included in this study**. The presence and number of *Dscam *orthologs and co-orthologs (*I. scapularis, I.s*.), *Dscam-hv *orthologs, and putative alternatively spliced exons within Immunoglobulins 2, 3 and 7, are indicated for each species. Grey horizontal boxes highlight the species for which our study adds some information: more specifically, black numbers show putative genes found or annotated in this study and black dashes show that we did not find *Dscam *orthologs/paralogs for that species. Grey numbers show the *Dscam *genes and *Dscam-hv *that were annotated or described prior to this study, although the number of Ig exon variants shown are those predicted by our HMMs. The genome of *L. vannamei *is not sequenced so the grey question marks indicate that we could not search for *Dscam-like *genes in this species. * indicates genes which were used to build HMMs to search for *Dscam *orthologs, and ‡ indicates genes which were used to build the *Dscam-hv *HMMs. § For reasoning why this is 47 and not 48, please see Additional file 2. *L. vannamei *alternatively spliced exon numbers are conservative estimates [26]. Vertical bars indicate the main taxa of interest in this study, where A stands for Arachnida. Species relationships from [29-35].

The protein domain structure of Dscam-hv in *D. melanogaster *[6] consists of ten Ig domains: the alternatively spliced exon 4 lies within Ig2, exon 6 within Ig3, and exon 9 comprises the whole of Ig7 (Figure 1C). There are also six fibronectin type III (FN) repeats, a transmembrane domain and a C-terminal cytoplasmic tail [6]. Orthologs of the *Dscam-hv *gene have been found in other pancrustaceans (Figure 2), including several insect [17,25] and crustacean species [10,20,26,27], and all have the same ectodomain protein structure as *D. melanogaster*. However, there is considerable variation in the total number of alternatively spliced exons in the *Dscam-hv *genes across the Pancrustacea [10,17,26,28], and there is also variation in the number of conserved exons. Therefore for simplicity, hereafter we will refer to the hypervariable exons using domain numbering as these are likely to be more conserved than exon number. The question then arises of whether one could reconstruct the history of duplications and deletions that have shaped *Dscam-hv *evolution, and infer the ancestral set of alternatively spliced exons that were present at the base of the arthropods, pancrustaceans and insects. Also, of particular importance to the question of the ubiquity of *Dscam-hv*, it remains unclear when alternative splicing as found in *Dscam-hv *arose [11]. It has been assumed that *Dscam-hv *was already present in the genome of the common ancestor of the Pancrustacea but it is not known whether it was already present in the common ancestor of the arthropods. To date the most closely related species to the pancrustaceans that has been examined for *Dscam-hv *is the nematode *C. elegans*, although evidence of multiple exon variants was not found [28], and Brites and co-workers [10] subsequently suggested that it lacks *Dscam *altogether. Similarly more distantly related taxa within Protostomia, such as a platyhelminth and a mollusc, appear not to have *Dscam-hv *[10,11].

In addition to the hypervariable *Dscam-hv *gene, three non-hypervariable *Dscam *genes, so-called *Dscam-like *genes have been described in *D. melanogaster *[36]. One such gene, *Dscam2*, has two alternatively spliced exons in Ig7 [37], whereas *Dscam3 *and *Dscam4 *have only one isoform each. *Dscam2 *and *Dscam3 *have also been ascribed neuronal roles [37,38]. The ancestor to at least one of the *Dscam-like *genes was already present in the common ancestor of the holometabolous insects (Figure 2), because *Dscam-like *genes have been predicted in the genomes of the honeybee *Apis mellifera *(AbsCAM [39]), the yellow fever mosquito *Aedes aegypti *(used in the *Dscam *gene phylogeny presented in [10]) and the red flour beetle *Tribolium castaneum *(NCBI gene prediction XP_967655.2). A first approximation of the evolution of the insect *Dscam *gene family including the aforementioned genes was described by Brites and co-workers [10], however, it is unclear whether all of these species contain orthologs to all three *Dscam-like *genes as found in *D. melanogaster*, and therefore also at what point the *Dscam *gene family diversified: were the three *Dscam-like *genes already present in the common ancestor of the holometabolous insects, of all insects, or of the pancrustaceans?

*Dscam *orthologs are also present in deuterostomes. Unlike its pancrustacean counterpart, vertebrate *DSCAM *has only two predicted isoforms [40]. *DSCAM *also plays a fundamental role in, *e.g*., axon guidance [41] and self-avoidance [42], but as of yet no immune function has been suggested. A further vertebrate *DSCAM *homolog has also been identified (*DSCAML1 *[43]). Phylogenetic inference shows that the *Dscam/DSCAM *genes in protostomes and deuterostomes cluster separately; it follows that separate duplication events may have occurred leading to the independent expansion of the *Dscam/DSCAM *families in both lineages [10]. It seems that taxa quite distant from the bilateria (i.e., a plant and a yeast species) do not contain *Dscam *orthologs [28], however, it is unknown whether *Dscam *exists in a more closely related group to the Bilateria, *e.g*., the Cnidaria.

In this paper we take a bioinformatics approach, constructing Hidden Markov Model profiles (HMMs [44,45]) to search within the genomes of species for putative *Dscam *genes and hypervariable alternatively spliced exons and then reconstruct phylogenetic relationships between these genes and exons, to examine the evolutionary history of the *Dscam *gene family and the evolution of the three extracellularly expressed alternatively spliced exon clusters. Specifically we examine i) whether *Dscam *orthologs exist outside the Bilateria, if this is the case, it would suggest the origin of the *Dscam *gene family lies further back than previously believed, ii) whether the origin of *Dscam-hv *could lie outside the Pancrustacea, iii) when the *Dscam-like *orthologs arose, iv) how many alternatively spliced exons of each exon cluster were present at the base of the Arthropoda, Pancrustacea and the Insecta and v) whether the three exon clusters evolved similarly.

## Methods

For a general overview of the workflow followed, and extra methodological details, please see Additional files 1 and 2, respectively.

### Hidden Markov models

The complex exon structure of *Dscam-like *and *Dscam-hv *genes (Figure 1), and the fact that the exon structure is highly variable across *Dscam *orthologs, makes an ortholog search via the reciprocal best blast hit or the reciprocal smallest distance method [46] difficult. This is because these algorithms use alignment methods to search for orthologs that cannot incorporate the large introns contained in the *Dscam *genes, especially as they are interspersed with short exons. We therefore used profile Hidden Markov Models (HMMs) [44,45,47,48] (Additional files 1 and 2) as our technique to search for homologous *Dscam *sequences. HMMs are powerful tools often used for annotating sequences, for example domains in a protein sequence, or exons and introns in a nucletide sequence. An HMM is based on a probabilistic model, which in a biological context is usually provided by a multiple sequence alignment. From this model, probabilities are inferred for how likely it is that a certain observation in another sequence is made because the sequence fits the model, or how likely it is that the observation is made despite the sequence not fitting the model. We used HMMs to annotate protein sequences, both full putative Dscam proteins and individual exons, based on an alignment of already known protein sequences for the exon or protein.

### Genomes searched and sequences used for tree constructions

i) To examine whether *Dscam *homologs exist outside the Bilateria we included two cnidarians, *Hydra magnipapillata *(Figure 2; genome version 1.0 [49]) and the starlet sea anemone *Nematostella vectensis *(Figure 2; genome version 1.0 [50]).

ii) To test whether *Dscam-hv *exists outside of the Pancrustacea we searched the only arachnid (draft version assembly IscaW1, gene set ID IscaW1.1) genome available to date, the deer tick, *Ixodes scapularis *[51].

iii) To estimate when the *Dscam-like *orthologs arose we searched for *Dscam-like *genes in *I. scapularis*, as well as within nine pancrustaceans: the water flea, *Daphnia pulex *(genome version 1.0 [52]), six representative holometabolous insects, the mosquito *Anopheles gambiae *(genome version 3.4 [53]), *Apis mellifera *(genome version 4.0 [54]), the silk moth *Bombyx mori *(genome version 2.0 [55]), the flies *D. melanogaster *(genome version 5.0 [9]) and *D. mojavensis *(genome version 1.0 [56]) *Tribolium castaneum *(genome version 2.0 [57]) and two hemimetabolous insects, the pea aphid *Acyrthosiphon pisum *(genome version 1.0 [58]) and the human body louse *Pediculus humanus *(genome version 1.0 [59]). To infer the relationships between *Dscam-hv *and *Dscam-like *genes we included *Dscam-hv *from the whiteleg shrimp *Litopenaeus vannamei *[26], as well as *DSCAM *sequences from the California purple sea urchin *Strongylocentrotus purpuratus*, the zebra fish *Danio rerio*, the chicken *Gallus gallus*, the gray short-tailed opossum *Monodelphis domestica *and the house mouse *Mus musculus*; and *DSCAML1 *sequences from the dog *Canis lupus familiaris *and the brown rat *Rattus norvegicus*.

iv) and v) To examine the number of alternatively spliced exons at the base of the Pancrustacea and the evolutionary histories of the three exon clusters, we searched the previously annotated *Dscam-hv *genes as well as our newly annotated *Dscam-hv *genes from the above nine pancrustaceans and the putative *Dscam *genes identified for *I. scapularis *for alternatively spliced exons and then used these in our phylogenetic analyses.

### Identification of, and protein predictions for, *Dscam-like *orthologs

To identify *Dscam-like *orthologs we constructed HMMs from seven already available *Dscam-like *sequences. From *D. melanogaster *this included *Dscam2 *(see Additional file 3 for the NCBI accession number of this and the following sequences), *Dscam3 *and *Dscam4*. Two genes from *D. mojavensis *that are orthologous to *Dscam3 *and *Dscam4*, and two further *Dscam-like *orthologs from *T. castaneum *and *A. mellifera*. The protein sequences were aligned using MUSCLE [60]. The exon structure of these genes is variable between species, therefore to construct the HMMs (using HMMER 2.3.2; http://hmmer.org/[44]) we identified ten highly conserved regions of the alignment (Additional file 4) that did not cross exon boundaries in any of the *D. melanogaster Dscam2, Dscam3 *or *Dscam4 *genes (Additional file 5). We translated the genomes of the arthropods *A. gambiae, A. mellifera, A. pisum, B. mori, D. pulex, D. mojavensis, I. scapularis, P. humanus *and *T. castaneum *as well as the cnidarians, *H. magnipapillata *and *N. vectensis *into six reading frames and the resulting protein sequences were searched for matches to the ten HMMs using the hmmpfam algorithm. We took a conservative approach and considered the prediction as a putative *Dscam *ortholog when six of the ten HMMs matched the scaffold in the correct order (for rationale see Additional files 4, 6, 7 and 8) and each HMM match reached an e-value of 0.001 or less. Furthermore, we did not predict the domain structures, thus some domains may not be present for some of the *Dscam-like *predictions. To obtain the complete sequence of the predicted proteins, the Fgenesh + algorithm [61] was used on the identified scaffolds, using the most closely related (species-wise) available *Dscam-like *sequence as an orthologous sequence. Every predicted protein sequence was aligned to the already-annotated *Dscam-like *sequences and manually checked for quality.

As a positive control and to explain the underlying rationale for only accepting putative *Dscam-like *orthologs if six of the ten HMMs (Additional files 4 and 6) matched, we constructed another set of HMMs without *D. mojavensis Dscam-like *genes and ran these HMMs on the translated *D. mojavensis *genome. *Dscam-hv *and three *Dscam-like *genes were found with all ten HMMS showing significant hits (Additional file 7). Using this data, we started to find non-*Dscam *genes when four of the HMMS matched our criteria (Additional file 7), but for five HMMs and above we only retrieved the three *Dscam-like *genes and *Dscam-hv*, and from Additional file 7 is apparent that five HMMs is in the plateau phase. We therefore suggest that we are more likely to have have false negatives rather than false positives as a result of using six HMMs and above as the cut-off value (see Additional file 8 for HMM results for all arthropods). As a negative control we created a random nucleotide 'genome'; this contained 55,000 scaffolds each consisting of 30,000 nucleotides (an order of magnitude larger than the *D. melanogaster *genome) translated into all six reading frames. When we searched this data set with the HMMs, it never yielded more than one hit per scaffold with an e-value of less than 0.01.

### Identification and annotation of *Dscam-hv *genes

To build the HMMs for the *Dscam-hv *genes, we aligned the complete *Dscam-hv *protein sequences from *A. mellifera *(see Additional file 3 for the NCBI accession number of this and the following sequences), *D. melanogaster *and *D. pulex *using MUSCLE and annotated all exon borders. The global alignment was divided into 37 smaller alignments, which covered nearly the whole gene, whilst ensuring that none of the HMMs included exon borders; we then used HMMER to construct the protein HMMs from these alignments (Additional file 9). We used these HMMs to annotate previously incompletely annotated *Dscam-hv *exons from sequences that were already available from the literature for *A. gambiae, B. mori *and *T. castaneum*. The nucleotide sequences were translated into three reading frames and each HMM searched for the best two hits within these three reading frames. The best hits were then assembled into the full mRNA and protein sequences. For the remaining species where *Dscam-hv *had not been already fully annotated (*A. pisum, I. scapularis *and *P. humanus*), the whole genomes were translated into all six reading frames. The 37 HMMs were used to search the translated genomes and candidate genes proposed when more than 10 of these HMMs matched well (an e-value lower than 0.01 because these HMMs were longer than for the *Dscam-like *genes). These scaffolds were treated in the same way as the already identified sequences from *A. gambiae, B. mori*, and *T. castaneum*. For one species, *A. pisum*, this method was able to identify two scaffolds, one containing the first third of *Dscam-hv *and the other that overlapped this scaffold and contained the remaining two thirds. The two scaffolds were manually edited together, resulting in the full gene sequence. We also ran the HMMs over the translated *H. magnipapillata *and *N. vectensis *genomes.

### Identification and annotation of *Dscam-hv *hypervariable exons

To identify and annotate the hypervariable exons in the *Dscam-hv *orthologs of *A. gambiae, A. pisum, B. mori, P. humanus *and *T. castaneum*, and to search for hypervariable exons in *I. scapularis*, HMMs of the previously annotated exon variants of *D. melanogaster, A. mellifera*, and *D. pulex *were constructed (see Additional files 2, 10, 11 and 12). Not all hypervariable exons were used to build the HMMs, as using many exons that are closely related could result in over-representation of these exons, and may have lead to non-detection of exon variants that are evolutionarily further away. Therefore, approximate trees (RAxML, 100 rapid bootstraps with GTR + Γ4) were built from all available sequences for each of Ig2, Ig3 and Ig7, and only one representative exon from each clade was used for the HMMs. As a positive control, these HMMs were tested against the intronic regions of species in which these exons have already been annotated and all previously annotated exon variants could be found. We focused on the exons that correspond to extracellular domains of the protein, i.e. 4, 6 and 9. Therefore the intronic regions between the exons homologous to *D. melanogaster *exons 3 and 5, 5 and 7, and 8 and 10 of the species mentioned above, were translated into six reading frames and searched using the HMMs. All six reading frames were used so that potential inversion events could also be detected. After inspecting the distribution of e-values among the matches (Additional file 13), a cut-off of 0.001 was chosen for the exons to be included in the rest of the analyses. Nucleotide sequences of putatively alternatively spliced exons were then retrieved from the intronic sequences to use for the phylogenies.

### Phylogeny of the *Dscam *gene family

An alignment of all known and newly-identified *Dscam-like *orthologs as well as all *Dscam-hv *genes and the Deuterostoma *DSCAM *orthologs was created using several methods. First, a complete protein alignment of all 44 sequences was created using MUSCLE. All Ig2, Ig3, and Ig7 orthologous regions were removed from the alignment. The resulting 3,096 amino acid positions-long alignment was then shortened to 47% of its original length using Gblocks, allowing for gap positions [62] in order to allow for a more accurate deep phylogeny without alignment mismatches obscuring these deep nodes, and to break down artifacts such as long branch attraction [63]. The final alignment encompassed 44 sequences and spanned 1467 positions, comprising of 1420 alignment patterns and 14.9% gaps and undetermined positions (Additional file 14). The LG protein substitution matrix [64] was identified as the best-suited evolutionary model for our data set using ProtTest [65]. From this alignment, phylogenetic inference was performed on a maximum likelihood framework using RAxML v7.2.8 [66] with the LG + Γ4 model and computing 1,000 bootstrap replicates starting with a random tree. Bayesian phylogenetic inference was performed in parallel using PhyloBayes 3.3 [67] and LG + Γ4 and the CAT approximation for profile mixture. Three independent Monte Carlo Markov chains were launched, allowed to reach stationary state, checked for convergence among them and the posterior distribution sampled every 100 values to collect 1,000 data points. Rogue taxa, as defined by Pattengale and co-workers [68] were identified with RAxMLv7.2.8 using the -f R option.

Both maximum likelihood and Bayesian approaches recovered sequences from arthropods and deuterostomes as being respectively monophyletic. Arthropod sequences clustered into seven well-supported clades, although their relative phylogenetic relationships could not be inferred with confidence. To test different alternative hypotheses for the evolution of the *Dscam *gene family relationships we constructed alternative topologies and performed the Shimodaira-Hasegawa test on them (SH-test [69]) (Additional file 15), as implemented in RAxMLv7.2.8. The SH-test allows one to compare different trees for a given alignment under a maximum likelihood framework, and to identify alternative topologies that may also be acceptable descriptions for the phylogenetic relationships among clades. The preferred tree was chosen by testing the equally good gene trees identified against the known evolutionary relationships among the corresponding organisms.

Time inference for the *Dscam *gene family was performed with PhyloBayes, fixing the topological relationships among clades to follow the best tree and the preferred tree (Additional file 15), using LG + Γ4 and the CAT approximation for profile mixture, a log normal model [70] for the relaxed clock, and a birth-death prior. The following species divergence times were used as calibrations in the clades in square brackets: fruit fly - mosquito, 238.5 - 295.4 Ma [*Dscam2*/*Dscam3*/*Dscam4*/*Dscam-hv*]; fruit fly - bee, 238.5 - 307.2 Ma [*Dscam2/Dscam3*]; human - zebrafish, 416.1 - 421.8 Ma [DSCAM]; human - cow, 95.3 - 113 Ma [DSCAM1] (divergence dates from [29]). Three Monte Carlo Markov chains were constructed and tested for convergence.

### Phylogeny of *Dscam-hv *exons

To construct the phylogenies of *Dscam-hv *exon clusters, the protein sequences of all putative exons found for that cluster were aligned together with one representative exon of an *I. scapularis *gene, which was identified in the preferred *Dscam *tree to serve as an outgroup for the arthropod genes. Only one exon sequence of *L. vannamei *was included for each of the three exon clusters, as the rest of the exon sequences were not yet publicly available. The protein sequences were aligned using MUSCLE, manually checked for quality and then back-translated into codon alignments using PAL2NAL [71]. At the nucleotide level the final alignments encompassed respectively for the Ig2, Ig3 and Ig7 exons, 106, 290 and 219 sequences; 197, 147 and 328 alignment patterns; and 22%, 18% and 21% gaps and completely undetermined positions (Additional files 16, 17 and18). Phylogenetic inference was performed with RAxML v7.2.6 at the nucleotide level using GTR + Γ4, without partitions and also introducing three partitions corresponding to the three codon positions. Two independent runs with 5,000 bootstrap cycles were calculated, starting with a random tree. Bayesian phylogenetic inference was also performed on the same alignment with PhyloBayes at the nucleotide level using GTR + Γ4 and the CAT approximation for profile mixture. Similar methods were used to infer relationships between the *D, melanogaster *and the *D. mojavensis Dscam-hv *exons only. In this case, at the nucleotide level the final alignments encompassed respectively for the Ig2, Ig3 and Ig7 exons, 25, 99 and 66 sequences; 148, 128 and 296 alignment patterns; and 6.8%, 8.9% and 0.6% gaps and completely undetermined positions (Additional files 19, 20 and 21).

## Results and discussion

We have identified novel putative members of the *Dscam *gene family across the Pancrustacea. These fell into six well-supported insect gene clades: five *Dscam-like *and one containing the *Dscam-hv *cluster. A seventh well-supported clade included the four *Dscam*-related genes present in the genome of the arachnid *I. scapularis*, each containing a maximum of only one Ig2, one Ig3 and one Ig7 variant. These findings suggest that the *Dscam *genes duplicated independently in the insects and an arachnid genome, which adds to the two independent *DSCAM *gene copies found in vertebrate genomes. Similarly to tropomyosin genes [72], a second form of gene expansion, tandem duplication, is also highly active in this gene family, and is thought to have resulted in numerous alternatively spliced exon variants. We also identified alternatively spliced exons within Ig2, Ig3 and Ig7 from various insect species, but the short sequence length and the limited amount of phylogenetic signal of the putative exons identified, precluded the reconstruction of deep relationships between the exon variants of all arthropods.

### Identification of, and protein predictions for, members of the *Dscam *family

Using probabilistic models (profile HMMs) derived from multiple protein sequence alignments of *Dscam-like *and of *Dscam-hv*, we searched across taxa for potential members of the *Dscam *family. Using our *Dscam-like *HMMs we were unable to detect any *Dscam-like *orthologs in either of the two cnidarian genomes (*N. vectensis *and *H. magnipapillata; *Figure 2; we found similar negative results for the *Dscam-hv *HMMs). This supports the hypothesis that the ancestor of the *Dscam *gene family appeared after the emergence of the Bilateria. However, it is possible that we did not find any orthologs because of gaps in the genome sequences, so it would be informative to re-run this search when genomes of better quality are available. It is worth noting that we have chosen conservative HMM cut-offs (Additional files 7 and 8), and we are aware that this may lead to negative results and may have excluded true orthologs if their phylogenetic signal was too weak or had been eroded.

Across the arthropod genomes searched, we found 20 putative *Dscam-like *genes, four of which were in *I. scapularis *(Figure 2; Additional file 6). All insect genomes contained between two and four putative *Dscam-like *genes. Similarly to Brites and co-workers [10], we could not find any putative genes closely related to *Dscam-like *in the *D. pulex *genome, although the authors did find two regions with homology to the *Dscam-like *genes that contained a different domain organisation [10] (see also unpublished data cited in [73]). We did not find any putative *Dscam-hv *genes in the tick genome, which supports the hypothesis that *Dscam-hv*, as found in the insects, whiteleg shrimp, and water flea, originated at the base of the Pancrustacea. To test this hypothesis further, it would be essential to scan more non-pancrustacean arthropod genomes when they become available, and also to examine the tick genome when it is fully assembled. We did, however, identify *Dscam-hv *for all of our pancrustacean species (Figure 2). No duplicated *Dscam-hv *genes were found in any of the genomes searched.

### Phylogeny of the *Dscam *gene family

**Figure 3:**
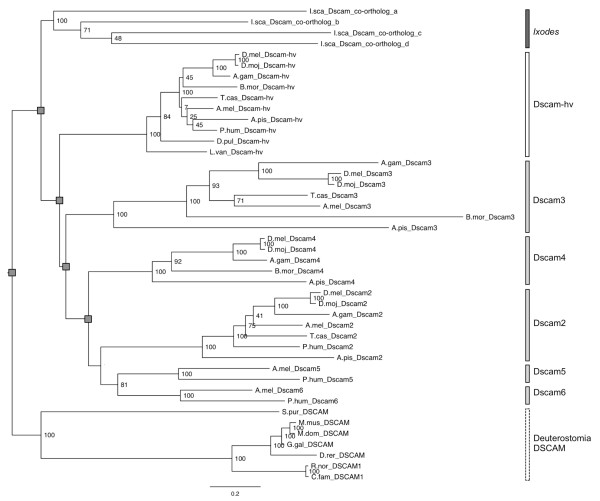
**Maximum likelihood (RAxML) phylogeny of the *Dscam*/DSCAM gene family**. Topology follows that of the preferred tree (Additional file 15), where grey squares indicate nodes that were fixed. Bootstrap values are shown at the non-fixed nodes. The vertical bars follow the bar colours of taxa written in black in Figure 2. The scale bar represents 0.2 substitutions per site.

With the newly identified sequences we aimed to reconstruct the phylogenetic relationships among the *Dscam-hv *and *Dscam-like *genes in arthropods, using deuterostome *Dscam *genes as an outgroup (Figure 2), and to incorporate time estimates in the Bayesian calculations by introducing priors with information from the fossil record. Both maximum likelihood and Bayesian phylogenetic reconstructions (Additional files 22 and 23), suggested that all of the *Dscam-hv *genes form one clade. Both methods also identified five other well-supported insect clades of *Dscam-like *genes, three of which clustered around one of the three *D. melanogaster Dscam-like *genes, and which we therefore named as *Dscam2, Dscam3 *and *Dscam4 *according to which of the three *D. melanogaster *genes they were orthologous to (Figure 2, Additional files 6, 22 and 23). The sequences from the *I. scapularis *genome clustered together to form one clade, and are therefore co-orthologs [74] with respect to the rest of *Dscam-hv *and *Dscam-like *clusters. Although the monophyly of each of the aforementioned seven large clades was clear, the phylogenetic position of certain individual taxa within the corresponding clade could not be clearly resolved, as several alternative branching positions could be identified to attain more than 0.95 posterior probability [68]. Such rogue taxa included co-orthologs a and c among the *Dscam *genes in *I. scapularis*, sequences from *T. castaneum, B. mori, A. pisum *and *A. mellifera *among the *Dscam-hv *genes, and sequences from *T. castaneum *and *A. mellifera *among the *Dscam2 *genes. In these instances, the inferred phylogenetic relationships among genes did not always match the evolutionary relationships among the corresponding organisms (Figures 2 &3). Problematic placements included *B. mori *(Figure 3, *Dscam-hv *and *Dscam3 *clades), where the predicted sequences contained sizeable gaps (Additional file 14), which could in part be due to the quality of the genome, as we indicated for our prediction of the *B. mori Dscam-hv *gene (Additional file 2). Furthermore, there was a sizeable gap in the predicted sequence of *T. castaneum Dscam3 *(Additional file 14), the placement of which also does not match the evolutionary relationships among the organisms.

The *Dscam2, Dscam3 *and *Dscam4 *clades all had multiple insect representatives (holometabolous and hemimetabolous species) suggesting that they may have arisen before radiation occurred within the insects. However, *Dscam5 *and *Dscam6 *contained representatives from only one hemimetabolous insect, *P. humanus*, and one holometabolous insect, *A. mellifera*: it seems unlikely that all of the other holometabolous insects have lost this gene. Our conservative cut-off for including putative genes in our phylogeny may have resulted in us missing representatives for these two clades from some of the other species. In the absence of topological constraints, but also when enforcing monophyly separately for each of the *Dscam-hv *and *Dscam-like *gene clades, the *Dscam3 *clade was basal to the rest of genes in the family (Additional files 22). Given the evolutionary relationships among arthropods (Figure 2), we hypothesised that the *I. scapularis *genes could be basal to those of the rest of the Pancrustacea. We tested this hypothesis by reconstructing a phylogeny in which *I. scapularis Dscam *co-orthologs were forced to be basal (Figure 3), the results of a SH-test showed that it was not significantly worse than the one in which the *Dscam3 *genes were basal (Additional file 15). We therefore propose that the ancestral *Dscam *gene was present in the genomes of the ancestral arthropods. The ancestral ortholog in the *I. scapularis *lineages underwent duplications and generated the *Dscam I. scapularis co-orthologs*, while the ortholog in the ancestral pancrustaceans also underwent duplication events that developed the family further. The alternative hypothesis would be that the monophyly of the four *Dscam I. scapularis *in-paralogs is an artifact caused by long-branch attraction and/or by convergence in amino acid preferences of the four genes in the *I. scapularis *genome. Only future research on basal arthropods will provide genomic data to test these exclusive hypotheses. Regarding crustaceans, the only gene prediction for the *D. pulex *genome corresponded to a *Dscam-hv *gene. This could be interpreted as the *Dscam-hv *clade to be basal to the rest of the *Dscam-like *ones. Indeed, a phylogenetic reconstruction enforcing such topology was not significantly worse than the best-known constrained tree, with *Dscam3 *being basal to the rest of pancrustacean *Dscam *clades (Additional file 15). The alternative hypothesis would either imply the selective loss of the *Dscam-like *gene in the *D. pulex *lineage, or the inability of our approach to detect it in its present form [see [10,73]].

**Figure 4:**
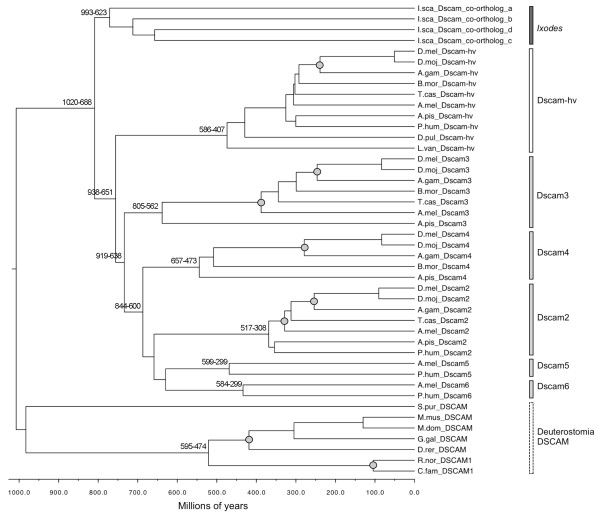
**Bayesian (PhyloBayes, relaxed clock) dated phylogeny of the *Dscam*/DSCAM gene family**. 95% confidence intervals for divergence times (millions of years) are shown next to the key nodes. The x-axis shows the time scale in millions of years. The topology of the main clades follows that of the preferred tree (Figure 3 & Additional file 15), with the addition of putative gene relationships within clades being fixed to follow species relationships shown in Figure 2. Nodes used for fossil calibrations are shown with a grey circle. The vertical bars follow the bar colours of taxa written in black in Figure 2.

To gain a deeper insight into the evolution of the *Dscam-like *and *Dscam-hv *clades, we dated our preferred tree using Bayesian reconstruction (Figure 4; see Additional file 24 for a reconstruction using the best tree). Our preferred dated tree where the *I. scapularis *clade was enforced as an outgroup (Figure 4) estimated that the *I. scapularis *clade split from the rest of the arthropods between 1020-688 million years ago (Ma), and the best tree estimate was between 928-642 Ma. These figures contain, but are on the edge of, Pisani's [75] 698.5 Ma mean divergence time estimate between Pancrustacea and Chelicerata. Our estimated date for the divergence of the deuterostomes and the protostomes (approximately 1000 Ma) is also quite old, but falls within other estimates, which range from 1,200 to 580 Ma [76]. Within the Insecta, the most recent common ancestor (mrca) respectively for the main *Dscam-like *clades (*Dscam2, Dscam3 *and *Dscam4*) was between 919-638 Ma (Figure 4). Furthermore it is noteworthy that in the *D. melanogaster *genome, the *Dscam2 *and the *Dscam4 *genes, closely related according to our reconstructions, are both encoded in chromosome 3 L, ca. 1 mega base pairs apart from one each other. The two additional basal clades, *Dscam5 *and *Dscam6*, appeared to have arisen from more recent duplication events (Figure 4 and Additional file 24). Our estimate for the mrca of the *Dscam-hv *(*D. pulex *and *L. vannamei*: around 586-407 Ma; Figure 4) fits quite well with other molecular estimates for the time of divergence between these species (respectively, 508-533 Ma and 540 Ma [77,78]). Furthermore our estimates for the basal splits within the insects, i.e., *Dscam-like *genes from hemimetabolous and holometabolous insects, were in the region of another estimate (Figure 4, Additional file 24; *e.g*. 355 Ma [30]).

More generally, our strict criteria for including a gene prediction as a putative *Dscam *ortholog (see methods), may have also resulted in us missing representatives from *Dscam-like *clades other than *Dscam5 *and *Dscam6*. For example, we identified no *P. humanus Dscam3 *and *Dscam4 *orthologs, and no *Dscam4 *ortholog for either *A. mellifera *or *T. castaneum *(Figure 2). It is possible that orthologs to those genes exist in the corresponding genomes, or alternatively, that after duplication events of the *Dscam-like *genes in the ancestor of the insects that these representatives were secondarily lost from some genomes. It is also unclear which mechanisms led to the generation of the copies of the ancestral *Dscam *gene in the genome of the ancestral insects in a relatively short period of time. It is indeed difficult to envisage how the duplication of a gene such as *Dscam-hv*, which spans almost 70 kilo base pairs in *D. melanogaster*, can be neutral and come at no cost. The conundrum about the forces driving the duplication and the advantages that condition the selection of the newly evolved genes make it difficult to classify these events according to the models of the evolution of gene duplications presented by Innan and Kondrashov [79].

### Identification and annotation of *Dscam-hv *hypervariable exons

Our HMMs found identical numbers of alternatively spliced exons to species where exon numbers had previously been predicted or shown to be expressed (see Additional file 2 for the three exceptions in Ig3). Using HMMs we annotated individual hypervariable exons from four insect species (Figure 2). For the four putative *I. scapularis Dscam co-orthologs *we did not find more than one Ig2, Ig3 or Ig7 ortholog within the regions of the genes searched. However, *Dscam co-ortholog a *appeared to have no Ig2 or Ig3 alternatively spliced variant (but see Additional file 2). We found no evidence of potential exon inversion events, i.e., putative exons were only found in three reading frames per genome.

### Phylogeny of *Dscam-hv *exons

An especially interesting aspect of the evolution of the *Dscam-hv *genes is the evolution of the alternatively spliced regions. In particular, is it possible to reconstruct how many alternatively spliced exons of each exon cluster were present at the base of the Pancrustacea and the Insecta, and did the three exon clusters evolved similarly? Using annotated exons from the literature and those predicted from our HMMs, we had 106 arthropod exons, ranging from 153 to 171 base pairs (bp) in length, with which to build the Ig2 phylogeny (Additional files 25 and 26). The phylogenetic relationships among exons could not be resolved with confidence, neither with maximum likelihood nor with Bayesian approaches (Additional files 25 and 26). In all cases the inferred trees displayed a star-like topology, without polarity, hindering the identification of groups of taxa or of exons that could belong together and making it unfortunately not possible to estimate the number of exon variants present at the base of the Arthropoda, Pancrustacea and Insecta. Indeed, for 58% (72/106) of the sequences the position in the tree varied largely with the region analysed and showed multiple possible insertion points to reach an accumulated 0.95 posterior probability. This is perhaps not surprising, since we were trying to resolve the relationships among 106 sequences with 197 alignment patterns. The fact that we were unable to reconstruct the deep relationships between the exon variants with confidence differs from the findings of Lee and co-workers [80], where they reconstructed the evolutionary history of each variable exon cluster across holometabolous insects and a crustacean species (*D. pulex*) and subsequently suggested that there were at least nine Ig2 exons in the insect ancestor and one exon present in the common ancestor of Pancrustacea. Our low bootstrap support values and posterior probabilities (Additional files 25 and 26) are likely the result of the relatively short lengths of a large number of exons combined with a long evolutionary timescale. Additional factors such as accelerated evolutionary rate, subfunctionalisation and/or gene conversion, may have further contributed to the erosion of the phylogenetic signal. Finally, a similar composition across non-orthologous exons due to amino acid and/or codon usage preferences (*e.g*., [81]) might complicate the analysis, and generate a misleading impression of monophyly. Examples of this putative convergence can be observed, for example, in Additional files 25 and 26, where exons predicted in the same genome tend to flock together. One methodological explanation for the difference between our study and that of Lee and co-workers [80] is that we used Bayesian and maximum likelihood methods whereas they used neighbour joining. However, such high resolution power for neighbour joining compared to maximum likelihood and to Bayesian analysis is unexpected, especially for short, highly divergent sequences, as is the case with these sequences.

**Figure 5:**
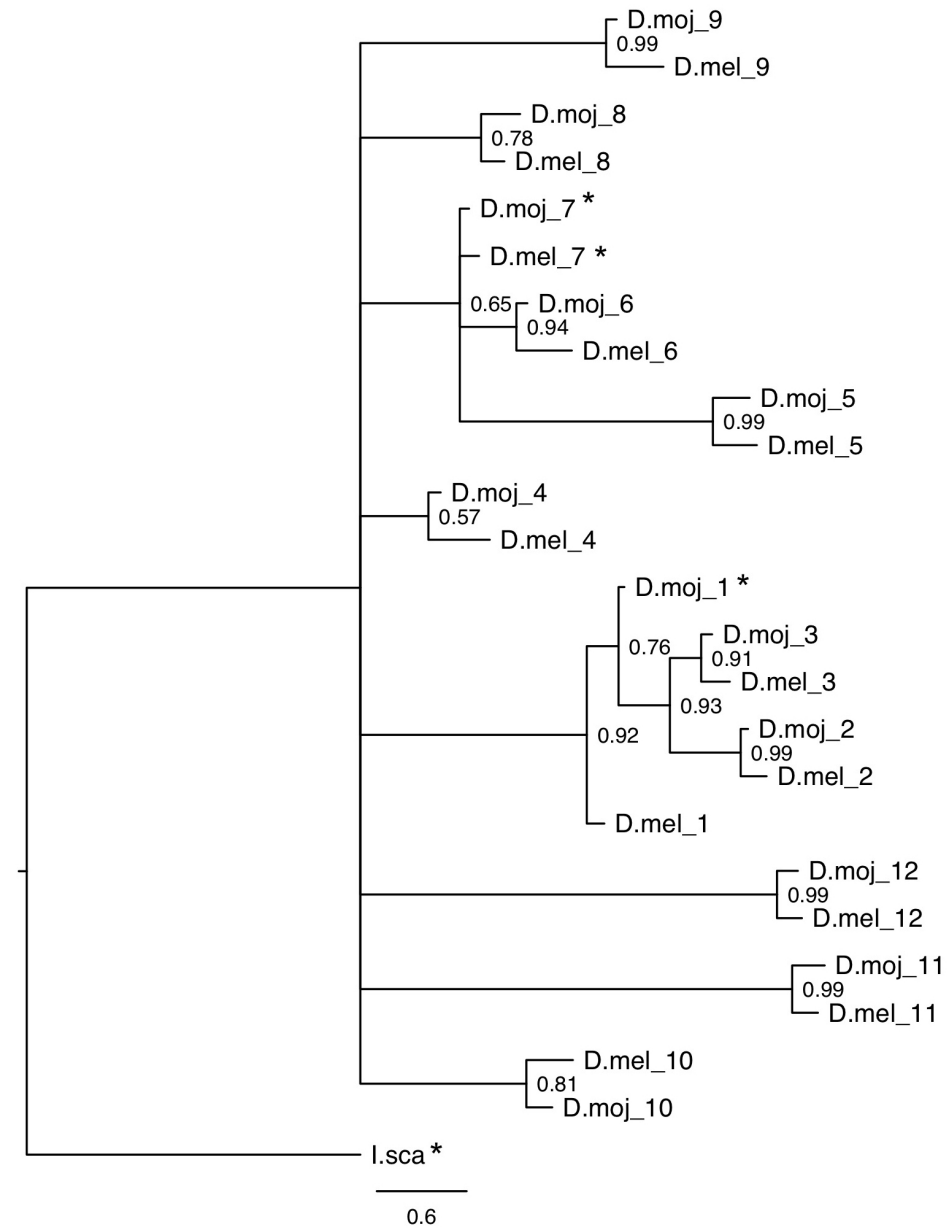
**Bayesian (PhyloBayes) phylogeny of hypervariable Ig2 variants (exon 4) from *Drosophila melanogaster *and *D. mojavensis***. A putative *Ixodes scapularis *Ig2 sequence was used as the outgroup. Bootstrap values are shown at the nodes. The scale bar represents 0.6 substitutions per site. Stars indicate rogue taxa, hence their phylogenetic positions cannot be inferred with confidence.

Because we could not infer the relationships between the exons over such an enormous evolutionary time scale, we tested whether we could find a better resolution at a scale of around 40-60 million years, i.e., the estimated divergence times for *D. melanogaster *and *D. mojavensis *[82,83]. Most exons from one species had clear orthologs in the other species (Figure 5 and Additional file 27), with no evolutionary events, whether losses or duplications, occurring since the species split. The number of rogue taxa decreased to 16% (4/25), but the position of the root and the fine relationships between orthologous pairs could not be resolved (Figure 5 and Additional file 27).

Our Ig3 phylogeny consisted of 290 arthropod exons, ranging from 102 to 132 bp in length, and the Ig7 phylogeny consisted of 219 arthropod exons, ranging from 243 to 312 bp in length. It was not possible to reconstruct a likely set of ancient exons for either of these exon clusters because of the low bootstrap support and posterior probabilities, and because a large proportion of the sequences were rogue taxa (64% and 46%, Ig3 and Ig7 respectively). Noticeably, many exons in the phylogeny are in species-specific clusters (Additional files 28, 29, 30). Again, it is difficult to say whether this clustering is due to multiple, parallel expansion events within a lineage, the current mainstream interpretation, because of the potentially confounding effect of codon usage variation across species [81] that may hinder phylogenetic reconstruction. As mentioned above, the clusters may therefore reflect nucleotide composition and not necessarily common ancestry. Similarly, Lee and co-workers [80] suggested limited ancestral reconstruction was possible, although they suggested that six Ig3 exons and two Ig7 exons were present in the insect ancestor.

While the total number of Ig3 exon variants in *D. melanogaster *only differs by two from that of *D. mojavensis*, a closer look at the overall phylogeny (Additional files 32 and 33) reveals that there were probably more than two duplication or deletion events in these lineages. This is in contrast to the Ig2 phylogeny, and underlines the fact that the accumulation of diversity in the Ig3 cluster occurred faster than in the Ig2 cluster. The orthologous Ig7 exons identified in *D. melanogaster *and *D. mojavensis *were consistent with one another across the two phylogenetic reconstruction methods (Additional files 34 and 35), they showed that the first half of the alternatively spliced exon variants (9.1 to 9.14) corresponded almost one for one to orthologs in both species, the exons in the middle region show slightly more complex across-species relationships indicative of duplications and losses, and towards the end of the exon cluster *D. melanogaster *exons 9.27 to 9.33 and their *D. mojavensis *counterparts also appear to be clear orthologs of one another. However, it was still not possible to resolve the deeper relationships between the orthologs.

Overall, the three hypervariable exon clusters appear to have experienced quite different evolutionary histories. While the Ig2 cluster is comparatively more conserved (consistent with [25,28]), Ig3 and Ig7 have experienced many more recent exon duplications and deletions and most of the diversity appears to have evolved since the split of the insects. All exon clusters seem to have evolved mainly via tandem duplications, and possibly fall into category III 'Diversifying selection' according to the models of gene-duplication evolution presented by Innan and Kondrashov [79]. No duplications of more than one exon could be found in the *Drosophila *analysis. The three exon clusters have also evolved independently of one another. The differences between the three exon clusters regarding their evolution are likely caused by different selection pressures on the exons. These could be due to different functions, for example in the nervous system versus in the immune system, but also due to their different position in the three-dimensional structure of the Dscam protein [84].

## Conclusions

Our analyses of the evolution of the *Dscam *family in the arthropods are compatible with the following evolutionary scenario. The genome of the arthropod ancestors already contained one copy of the ancestral *Dscam *gene. In the arachnid *I. scapularis*, gene duplication and diversification generated the four extant copies (co-orthologs) of the *Dscam *gene, which are exclusive to this lineage. In the pancrustaceans, a basal duplication event generated the ancestral *Dscam-hv *and the ancestor of the rest of the *Dscam-like *clades. The ancestral *Dscam-like *gene underwent a further series of between two and four duplication events in the lineage of the insects. In the *Dscam-hv *lineage, the expansion of alternatively spliced exons in the Ig2, Ig3 and Ig7 domains via duplication started early, or immediately after, the emergence of the ancestral *Dscam-hv *genes in pancrustaceans, and seems to be basal to this clade. There seems thus to exist an adaptive value in this broadening of the *Dscam-hv *transcript/protein repertoire that may have been available through neofunctionalisation after gene duplication. Such new function(s) could be related to the differentiation between self and non-self, as evidenced in their role in the nervous system and in their putative role in immunity. If it is true that the hypervariability in *Dscam-hv *arose after the split between arachnids and insects, one may wonder whether such new roles correspond to new solutions to old problems, also faced by arachnids and solved by other means, or whether they correspond to new functions that are exclusive to insects. A future search on more genomes with better quality will help fill the evolutionary gaps and clear the oddities that remain unexplained. Finally, only experimentation will enable us to solve questions about whether the putative *Dscam-like *genes are expressed and what their functions are, and to understand more about the role of alternative splicing of the hypervariable exons in *Dscam-hv *across the arthropods.

## Authors' contributions

SAOA participated in the conception and design of the study, participated in analyses and drafted the manuscript. RF built the HMMs, identified the putative *Dscam *sequences, and participated in building the phylogenetic trees. JK participated in the conception of the study and helped to draft the manuscript. IGB participated in the conception and design of the study, performed the phylogenetic and statistical analyses, and helped to draft the manuscript. All authors read and approved the manuscript

## Supplementary Material

Additional file 1 **An overview of the workflow followed for the HMM construction and use, and for the gene predictions**. Click here for file

Additional file 2 **Supplementary methods**. Click here for file

Additional file 3 **Genbank (NCBI) accession numbers for the known and putative *Dscam-hv *and *Dscam-like *genes used in the overall phylogenies**. Click here for file

Additional fie 4 **Information regarding the HMMs for the *Dscam-like *gene search**. Including the HMM identities, the lengths of each of the HMMs in amino acids, and the HMM amino acid start position relative to the *D. melanogaster Dscam2 *sequence (total length 2,040 aa). Click here for file

Additional file 5 ***Dscam-like *HMM**. Click here for file

Additional file 6 **Putative *Dscam-like *orthologs/co-orthologs and the number of Hidden Markov Models positively identified for each ortholog/co-ortholog**. Grey boxes indicate an HMM hit with an e-value ≤ 0.001. White boxes indicate an e-value greater than 0.001 or no match. The identities of the *Dscam-like *genes were assigned according to the phylogenetic tree in Figure 3. Click here for file

Additional file 7 ***D. mojavensis *HMM results**. Results for the *Dscam-like *HMMs built from *A. mellifera, D. melanogaster *and *T. castaneum *and run against the translated *D. mojavensis *genome. For a total of five 'matching' HMMs and above we found only four hits in the genome (i.e. four genes: one *Dscam-hv *and three *Dscam-like*), which had HMMs with significant hits in the correct order. The conservative cut-off value, which was subsequently used when searching other species for *Dscam-like *genes, is shown as a dashed line, i.e. a minimum of six HMMs had to match the sequence in the correct order and all with an e-value below 0.001. Click here for file

Additional file 8 **Arthropod HMM results**. Number of hits for the *Dscam-like *HMMs built from *A. mellifera, D. melanogaster, D. mojavensis *and *T. castaneum *and run against the translated genomes of *A. gambiae, A. mellifera, A. pisum, B. mori, D. pulex, I. scapularis, P. humanus humanus *and *T. castaneum*. The cut-off value is shown as a dashed line. Click here for file

Additional file 9 ***Dscam-hv *HMM**. Click here for file

Additional file 10 **Ig2 HMM**. Click here for file

Additional file 11 **Ig3 HMM**. Click here for file

Additional file 12 **Ig7 HMM**. Click here for file

Additional file 13 **E-value distribution among putative hypervariable exons found across all arthropod species using our HMMs**. The vertical dashed line marks the cut-off e-value of 0.0001. Click here for file

Additional file 14 **Amino acid alignment of putative Dscam gene family members**. The complete protein alignment of 44 sequences was created using MUSCLE. All Ig2, Ig3 and Ig7 orthologous regions were removed from the alignment. The resulting alignment was shortened using Gblocks. Click here for file

Additional file 15 **Tests of alternative tree topologies**. The "best" tree (top; Additional files 22 &23) was tested against alternative hypotheses for the relationships between different *Dscam *clades by constructing alternative topologies (bottom two trees) and performing the Shimodaira-Hasegawa test. Neither of the two alternative topologies was significantly worse than the "best" tree at the 1% level. Click here for file

Additional file 16 **Nucleotide alignment of all arthropod Ig2 variants**. Click here for file

Additional file 17 **Nucleotide alignment of all arthropod Ig3 variants**. Click here for file

Additional file 18 **Nucleotide alignment of all arthropod Ig7 variants**. Click here for file

Additional file 19 **Nucleotide alignment of all *D. melanogaster *and *D. mojavensis *Ig2 variants**. Click here for file

Additional file 20 **Nucleotide alignment of all *D. melanogaster *and *D. mojavensis *Ig3 variants**. Click here for file

Additional file 21 **Nucleotide alignment of all *D. melanogaster *and *D. mojavensis *Ig7 variants**. Click here for file

Additional file 22 **Maximum likelihood (RAxML) phylogeny of the *Dscam*/DSCAM gene family, resulting in the best tree **(Additional files 15 and 23). Bootstrap values (out of 100) are shown at the nodes. The vertical bars to the right are the same as in Figures 3 and 4 and follow the taxa colour codes in Figure 2. The scale bar represents 0.2 substitutions per site. Click here for file

Additional file 23 **Bayesian (PhyloBayes) phylogeny of the *Dscam*/DSCAM gene family, resulting in the best tree **(Additional file 15 & Additional file 22). Posterior probabilities are shown at the nodes. The vertical bars to the right are the same as in Figures 3 and 4 and follow the taxa colour codes in Figure 2. The scale bar represents 0.4 substitutions per site. Click here for file

Additional file 24 **Bayesian (PhyloBayes, relaxed clock) dated phylogeny of the *Dscam*/DSCAM gene family**. 95% confidence intervals for divergence times (millions of years) are shown next to the key nodes. The x-axis shows the time scale in millions of years. The topology follows that of the original best tree (see Additional file 15, 22 and Additional file 23). Nodes used for fossil calibrations are shown with a grey circle, for details see materials and methods. The vertical bars follow the bar colours of taxa written in black in Figure 2. Click here for file

Additional file 25 **Bayesian (PhyloBayes) phylogeny of all hypervariable Ig2 variants across the arthropods**. A putative *Ixodes scapularis *Ig2 sequence is the outgroup. Bootstrap values are shown at the nodes. The scale bar represents 0.4 substitutions per site. Click here for file

Additional file 26 **Maxiumum likelihood (RAxML) phylogeny of all hypervariable Ig2 variants across the arthropods**. A putative *Ixodes scapularis *Ig2 sequence is the outgroup. Bootstrap values are shown at the nodes. The scale bar represents 0.3 substitutions per site. Click here for file

Additional file 27 **Maximum likelihood (RAxML) phylogeny of hypervariable Ig2 variants (exon 4) from *Drosophila melanogaster *and *D. mojavensis***. A putative *Ixodes scapularis *Ig2 sequence is the outgroup. Bootstrap values are shown at the nodes. The scale bar represents 0.3 substitutions per site. Click here for file

Additional file 28 **Bayesian (PhyloBayes) phylogeny of all hypervariable Ig3 variants across the arthropods**. A putative *Ixodes scapularis *Ig3 sequence is the outgroup. Bootstrap values are shown at the nodes. The scale bar represents 0.3 substitutions per site. Click here for file

Additional file 29 **Maxiumum likelihood (RAxML) phylogeny of all hypervariable Ig3 variants across the arthropods**. A putative *Ixodes scapularis *Ig3 sequence is the outgroup. Bootstrap values are shown at the nodes. The scale bar represents 0.4 substitutions per site. Click here for file

Additional file 30 **Bayesian (PhyloBayes) phylogeny of all hypervariable Ig7 variants across the arthropods**. A putative *Ixodes scapularis *Ig7 sequence is the outgroup. Bootstrap values are shown at the nodes. The scale bar represents 0.5 substitutions per site. Click here for file

Additional file 31 **Maxiumum likelihood (RAxML) phylogeny of all Ig7 variants across the arthropods**. A putative *Ixodes scapularis *Ig7 sequence is the outgroup. Bootstrap values are shown at the nodes. The scale bar represents 0.6 substitutions per site. Click here for file

Additional file 32 **Bayesian (PhyloBayes) phylogeny of hypervariable Ig 3 variants (exon 6) from *Drosophila melanogaster *and *D. mojavensis***. A putative *Ixodes scapularis *Ig3 sequence is the outgroup. Bootstrap values are shown at the nodes. The scale bar represents 1 substitution per site. Click here for file

Additional file 33 **Maximum likelihood (RAxML) phylogeny of hypervariable Ig3 variants (exon 6) from *Drosophila melanogaster *and *D. mojavensis***. A putative *Ixodes scapularis *Ig3 sequence is the outgroup. Bootstrap values are shown at the nodes. The scale bar represents 0.6 substitutions per site. Click here for file

Additional file 34 **Bayesian (PhyloBayes) phylogeny of hypervariable Ig7 variants (exon 9) from *Drosophila melanogaster *and *D. mojavensis***. A putative *Ixodes scapularis *Ig7 sequence is the outgroup. Bootstrap values are shown at the nodes. The scale bar represents 2 substitutions per site. Click here for file

Additional file 35 **Maximum likelihood (RAxML) phylogeny of hypervariable Ig7 variants (exon 9) from *Drosophila melanogaster *and *D. mojavensis***. A putative *Ixodes scapularis *Ig7 sequence is the outgroup. Bootstrap values are shown at the nodes. The scale bar represents 0.3 substitutions per site. Click here for file
