# Supplementary material for: The evolution of Dscam genes across the arthropods
Source: BMC Evol Biol. 2012 Apr 13;12:53. doi: 10.1186/1471-2148-12-53 (PMC3364881; doi:10.1186/1471-2148-12-53)
Supplement: Additional file 1 — An overview of the workflow followed for the HMM construction and use, and for the gene predictions. [file 1471-2148-12-53-S1.DOC]

**
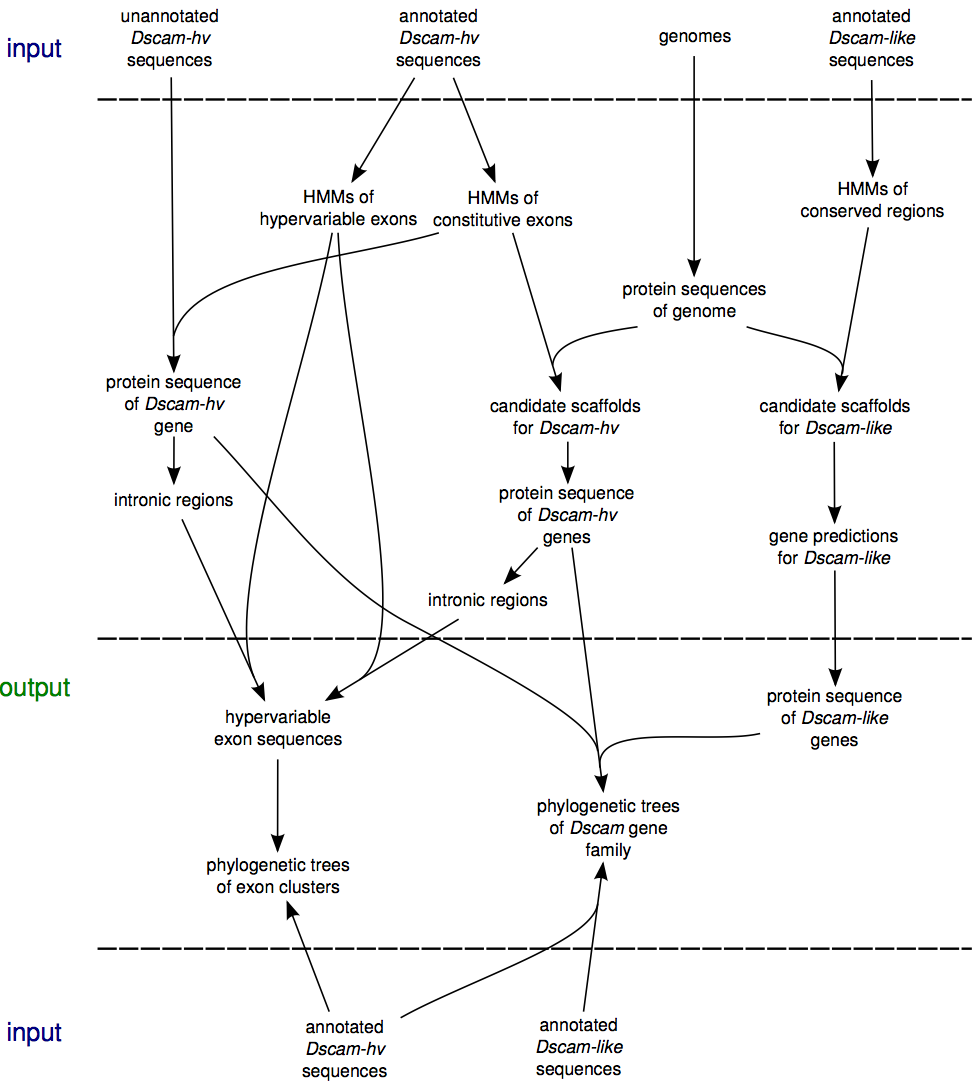
**

**Additional file 1.** An overview of the workflow followed for the HMM construction and use, and for the gene predictions.
