# Supplementary material for: The evolution of Dscam genes across the arthropods
Source: BMC Evol Biol. 2012 Apr 13;12:53. doi: 10.1186/1471-2148-12-53 (PMC3364881; doi:10.1186/1471-2148-12-53)
Supplement: Additional file 2 — Supplementary methods. [file 1471-2148-12-53-S2.DOC]

**Additional file 2 – Supplementary methods**

Identification and annotation of *Dscam-hv* genes

As a negative control, we used the same random nucleotide ‘genome’ as used for the *Dscam-like* HMMs. Once again, this search never yielded more than one hit per scaffold with an e-value of less than 0.01. The insect *Dscam-hv* were of a similar length to *D. melanogaster*, the only exception to this was *B. mori*. In this species the gene was located on a scaffold that ended after approximately two thirds of the protein sequence and we could not find the rest of the gene in the remaining scaffolds. Given the conservation of all modules within *Dscam-hv*, it is likely that the absence of the 3’ terminus of the sequence in *B. mori* is due to an incomplete genome sequence rather than a sequence loss.

Annotation of *Dscam-hv* hypervariable exons

The HMMs we use appear to be relatively robust, for example the genome of the leafcutting ant *Atta cephalotes* genome recently became available (Suen et al., 2011), and using our Ig2, 3 and 7 HMMs were were able to detect 8 x Ig2, 31 x Ig3, and 17 x Ig7 variants. This suggests that compared to its closest relative (*A. mellifera*) from which it diverged around 164 MYA (Brady, Larkin and Danforth 2009), *A. cephalotes* has the same number of exon variants, except for Ig3, where there is a difference of 14.

In general the number of exons we predicted with our HMMs is remarkably consistent with the numbers available in the literature. However, it is notable that we have slightly different predicted numbers of variable exons for Ig3 for three species compared to what has already been found in the literature. For *T. castaneum*: our HMM prediction gave 27 exons, Watson et al. (2005) and Lee et al. (2010) found 28 exons; *D. mojavensis*: our HMM prediction gave 51 exons, Lee et al. (2010) found 50; *D. melanogaster*: our HMM prediction gave 47 exons, Watson et al. (2005) found 48; the latter is most probably due to the somewhat less conserved sequence of Watson et al’s (2005) exon 6.11, which incidentally does not seem to be expressed (Neves et al., 2004; Watson et al., 2005). Our HMM for Ig7 also detected two approximately half-length putative variants for *B. mori,* which were found in different reading and were partially overlapping. One half-length variant contained a sequence similar to the N-terminal half of Ig7 and the other variant a sequence similar to the C-terminal half of Ig7. In order to be conservative we omitted this putative variant from our phylogenies. The alternatively spliced exon cluster in Ig2 showed the lowest number of variants across the pancrustacean species (12.4 ± 1.3; mean ± 1 s.e.), Ig7 had the second highest (22.6 ± 3.1) and Ig3 the highest and most variable number (31.4 ± 3.8).

Our HMMs did not detect Ig2 or Ig3 variants for *I. scapularis co-ortholog a* (DS_632703). The lack of the former could be due to incomplete genome sequencing: in the sequence searched, 1,694 base pairs out of a total of 9,491 base pairs were unsequenced (i.e. ‘N’s). This was not however, the case for Ig3. The HMM for Ig7 also detected two putative variants for *I. scapularis co-ortholog c* (DS_922315), one with an e-value of 7.4e-19 and a second of 9.8e-8. The latter however, was only 60% of the length of the HMM for this immunoglobulin.

**Literature cited in the supplementary material**

Brady SG, Larkin L, Danforth BN. 2009. Bees, ants, and stinging wasps (Aculeata). In:

Hedges SB, Kumar S, editors. The timetree of life. Oxford University Press. P. 264-269.

Eddy SR. 1996. Hidden Markov models. Curr. Opin. Struct. Biol. 6:361-365.

Eddy SR. 1998. Profile hidden Markov models. Bioinformatics. 14:755-763.

Eddy SR. 2004. What is a hidden Markov model? Nat. Biotechnol. 22:1315-1316.

Lartillot N, Lepage T, Blanquart S. 2009. PhyloBayes 3: a Bayesian software package for

phylogenetic reconstruction and molecular dating. Bioinformatics. 25:2286-2288.

Lee C, Kim N, Roy M, Graveley BR. 2010. Massive expansions of Dscam splicing diversity

via staggered homologous recombination during arthropod evolution. RNA. 16:91-105.

Neves G, Zucker J, Daly M, Chess A. 2004. Stochastic yet biased expression of multiple

Dscam splice variants by individual cells. Nature Genetics. 36:240-246.

Stamatakis A, Ludwig T, Meier H. 2005. RaxML-III: a fast program for maximum

likelihood-based inference of large phylogenetic trees. Bioinformatics. 21:456-463.

Suen G, Teiling C, Li L, et al. (49 co-authors). 2011. The genome sequence of the leaf-cutter ant *Atta cephalotes* reveals insights into its obligate symbiotic lifestyle. PLoS Genetics 7: e1002007. *(Please note that 49 includes all authors of the article.)*

Watson FL, Püttmann-Holgado R, Thomas F, Lamar DL, Hughes M, Kondo M, Rebel VI, Schmucker D. 2005. Extensive diversity of Ig-superfamily proteins in the immune system of insects. Science. 309:1874-1878.
