# Supplementary material for: The evolution of Dscam genes across the arthropods
Source: BMC Evol Biol. 2012 Apr 13;12:53. doi: 10.1186/1471-2148-12-53 (PMC3364881; doi:10.1186/1471-2148-12-53)
Supplement: Additional file 3 — Genbank (NCBI) accession numbers for the known and putative Dscam-hv and Dscam-like genes used in the overall phylogenies. [file 1471-2148-12-53-S3.DOC]

**Additional file 3.** NCBI accessions for the known and putative *Dscam-hv* and *Dscam-like* genes used in the overall phylogenies.

| **Species** | **Gene ID in figures** | **NCBI accession (nt or protein)** |
| --- | --- | --- |
| *D. melanogaster* | D.mel_Dscam2 | NP_729223.2 |
|  | D.mel_Dscam3 | AAF55426.2 |
|  | D.mel_Dscam4 | NP_001137908.1 |
|  | D.mel_Dscam-hv | AAF71926.1 |
| *D. mojavensis* | D.moj_Dscam2 | XP_002009090.1 |
|  | D.moj_Dscam3 | XP_001999910.1 |
|  | D.moj_Dscam4 | XP_002012087.1 |
|  | D.moj_Dscam-hv | XP_002006063.1 |
| *A. gambiae* | A.gam_Dscam2 | AAAB01008948.1 |
|  | A.gam_Dscam3 | AAAB01008900.1 |
|  | A.gam_Dscam4 | AAAB01008807.1 |
|  | A.gam_Dscam-hv | NT_078267.5|:12419441-12470984 |
| *B. mori* | B.mor_Dscam3 | nscaf2822* |
|  | B.mor_Dscam4 | nscaf2204* |
|  | B.mor_Dscam-hv | DQ141103.1 |
| *T. castaneum* | T.cas_Dscam2 | XP_967655.2 |
|  | T.cas_Dscam3 | AAJJ01000913.1 |
|  | T.cas_Dscam-hv | NW_001092892.1 |
| *A. mellifera* | A.mel_Dscam2 | BAF03050.1 |
|  | A.mel_Dscam3 | NW_001253314.1 |
|  | A.mel_Dscam5 | NW_001253269.1 |
|  | A.mel_Dscam6 | NW_001253268.1 |
|  | A.mel_Dscam-hv | NP_001014991.1 |
| *A. pisum* | A.pis_Dscam2 | NW_001934231.1 |
|  | A.pis_Dscam3 | NW_001925066.1 |
|  | A.pis_Dscam4 | NW_001920015.1 |
|  | A.pis_Dscam-hv | NW_001935353.1 / NW_001934231.1 |
| *P. humanus* | P.hum_Dscam2 | AAZO01005217.1 |
|  | P.hum_Dscam5 | AAZO01006957 |
|  | P.hum_Dscam6 | AAZO01000378.1 |
|  | P.hum_Dscam-hv | AAZO01007341.1 |
| *L. vannamei* | L.van_Dscam-hv | ACZ26466.1 |
| *D. pulex* | D.pul_Dscam-hv | EU307884.1 |
| *I. scapularis* | I.sca_Dscam_co-ortholog_a | DS632703.1 |
|  | I.sca_Dscam_co-ortholog_b | DS979349.1 |
|  | I.sca_Dscam_co-ortholog_c | DS922315.1 |
|  | I.sca_Dscam_co-ortholog_d | DS825389.1 |
| *D. rerio* | D.rer_DSCAM | AAT36313.1 |
| *G. gallus* | G.gal_DSCAM | XP_416734.2 |
| *M. domestica* | M.dom_DSCAM | XP_001370653.2 |
| *C. familiaris* | C.fam_DSCAM1 | XM_546506.3 |
| *M. musculus* | M.mus_DSCAM | NP_112451.1 |
| *R. norvegicus* | R.nor_DSCAM1 | NP_001101611.1 |
| *S. purpuratus* | S.pur_DSCAM | XP_793690.2 |

*Sequences from http://silkworm.genomics.org.cn/silkdb/
