# Supplementary material for: The evolution of Dscam genes across the arthropods
Source: BMC Evol Biol. 2012 Apr 13;12:53. doi: 10.1186/1471-2148-12-53 (PMC3364881; doi:10.1186/1471-2148-12-53)
Supplement: Additional fie 4 — Information regarding the HMMs for the Dscam-like gene search. Including the HMM identities, the lengths of each of the HMMs in amino acids, and the HMM amino acid start position relative to the D. melanogaster Dscam2 sequence (total length 2,040 aa). [file 1471-2148-12-53-S4.DOC]

**Additional file 4.** Information regarding the HMMs for the *Dscam-like* gene search: the HMM identities, the lengths of each of the HMMs in amino acids, and the HMM amino acid start position relative to the *D. melanogaster* *Dscam2* sequence (total length 2,040 aa).

| **HMM id** | **Length (number of amino acids)** | **HMM start position relative to *D.melanogaster* *Dscam2*** |
| --- | --- | --- |
| 1 | 53 | 129 |
| 2 | 45 | 421 |
| 3 | 43 | 520 |
| 4 | 39 | 706 |
| 5 | 75 | 971 |
| 6 | 48 | 1110 |
| 7 | 56 | 1165 |
| 8 | 61 | 1284 |
| 9 | 42 | 1497 |
| 10 | 35 | 1690 |
