# Supplementary material for: The evolution of Dscam genes across the arthropods
Source: BMC Evol Biol. 2012 Apr 13;12:53. doi: 10.1186/1471-2148-12-53 (PMC3364881; doi:10.1186/1471-2148-12-53)
Supplement: Additional file 6 — Putative Dscam-like orthologs/co-orthologs and the number of Hidden Markov Models positively identified for each ortholog/co-ortholog. Grey boxes indicate an HMM hit with an e-value ≤ 0.001. White boxes indicate an e-value greater than 0.001 or no match. The identities of the Dscam-like genes were assigned according to the phylogenetic tree in Figure 3. [file 1471-2148-12-53-S6.DOC]

**Additional file 6.** Putative *Dscam-like* orthologs / co-orthologs and the number of Hidden Markov Models positively identified for each ortholog / co-ortholog. Grey boxes indicate an HMM hit with an e-value ≤ 0.001. White boxes indicate an e-value greater than 0.001 or no match. The identities of the *Dscam-like* genes were assigned according to the phylogenetic tree in fig. 3.

|  |  | **HMM number** | | | | | | | | | |
| --- | --- | --- | --- | --- | --- | --- | --- | --- | --- | --- | --- |
| **Species** | ***Dscam-like* gene id** | **1** | **2** | **3** | **4** | **5** | **6** | **7** | **8** | **9** | **10** |
| *A. pisum* | Dscam2 |  |  |  |  |  |  |  |  |  |  |
|  | Dscam3 |  |  |  |  |  |  |  |  |  |  |
|  | Dscam4 |  |  |  |  |  |  |  |  |  |  |
| *A. gambiae* | Dscam2 |  |  |  |  |  |  |  |  |  |  |
|  | Dscam3 |  |  |  |  |  |  |  |  |  |  |
|  | Dscam4 |  |  |  |  |  |  |  |  |  |  |
| *A. mellifera* | Dscam3 |  |  |  |  |  |  |  |  |  |  |
|  | Dscam5 |  |  |  |  |  |  |  |  |  |  |
|  | Dscam6 |  |  |  |  |  |  |  |  |  |  |
| *B. mori* | Dscam3 |  |  |  |  |  |  |  |  |  |  |
|  | Dscam4 |  |  |  |  |  |  |  |  |  |  |
| *D. mojavensis* | Dscam2 |  |  |  |  |  |  |  |  |  |  |
| *I. scapularis* | Dscam_co-ortholog_a |  |  |  |  |  |  |  |  |  |  |
|  | Dscam_co-ortholog_c |  |  |  |  |  |  |  |  |  |  |
|  | Dscam_co-ortholog_b |  |  |  |  |  |  |  |  |  |  |
|  | Dscam_co-ortholog_d |  |  |  |  |  |  |  |  |  |  |
| *P. humanus* | Dscam2 |  |  |  |  |  |  |  |  |  |  |
|  | Dscam5 |  |  |  |  |  |  |  |  |  |  |
|  | Dscam6 |  |  |  |  |  |  |  |  |  |  |
| *T. castaneum* | Dscam3 |  |  |  |  |  |  |  |  |  |  |
