# Supplementary material for: The evolution of Dscam genes across the arthropods
Source: BMC Evol Biol. 2012 Apr 13;12:53. doi: 10.1186/1471-2148-12-53 (PMC3364881; doi:10.1186/1471-2148-12-53)
Supplement: Additional file 7 — D. mojavensis HMM results. Results for the Dscam-like HMMs built from A. mellifera, D. melanogaster and T. castaneum and run against the translated D. mojavensis genome. For a total of five 'matching' HMMs and above we found only four hits in the genome (i.e. four genes: one Dscam-hv and three Dscam-like), which had HMMs with significant hits in the correct order. The conservative cut-off value, which was subsequently used when searching other species for Dscam-like genes, is shown as a dashed line, i.e. a minimum of six HMMs had to match the sequence in the correct order and all with an e-value below 0.001. [file 1471-2148-12-53-S7.DOC]

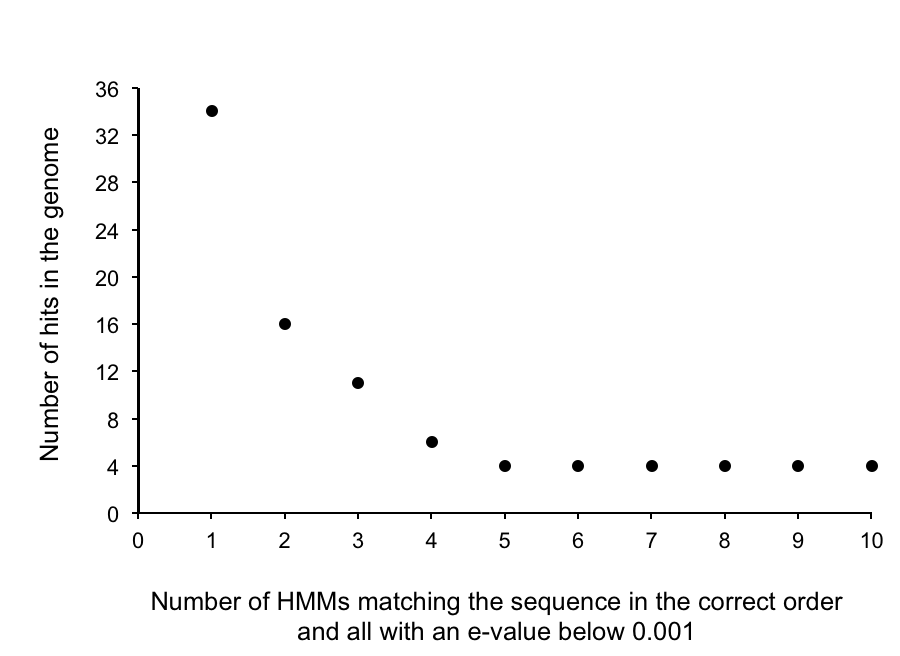


**Additional file 7.** *D. mojavensis* HMM results. Results for the *Dscam-like* HMMs built from *A. mellifera, D. melanogaster* and *T. castaneum* and run against the translated *D. mojavensis* genome. For a total of five ‘matching’ HMMs and above we found only four hits in the genome (i.e. four genes: one *Dscam-hv* and three *Dscam-like*), which had HMMs with significant hits in the correct order. The conservative cut-off value, which was subsequently used when searching other species for *Dscam-like* genes, is shown as a dashed line, i.e. a minimum of six HMMs had to match the sequence in the correct order and all with an e-value below 0.001.
