# Supplementary material for: The evolution of Dscam genes across the arthropods
Source: BMC Evol Biol. 2012 Apr 13;12:53. doi: 10.1186/1471-2148-12-53 (PMC3364881; doi:10.1186/1471-2148-12-53)
Supplement: Additional file 8 — Arthropod HMM results. Number of hits for the Dscam-like HMMs built from A. mellifera, D. melanogaster, D. mojavensis and T. castaneum and run against the translated genomes of A. gambiae, A. mellifera, A. pisum, B. mori, D. pulex, I. scapularis, P. humanus humanus and T. castaneum. The cut-off value is shown as a dashed line. [file 1471-2148-12-53-S8.DOC]

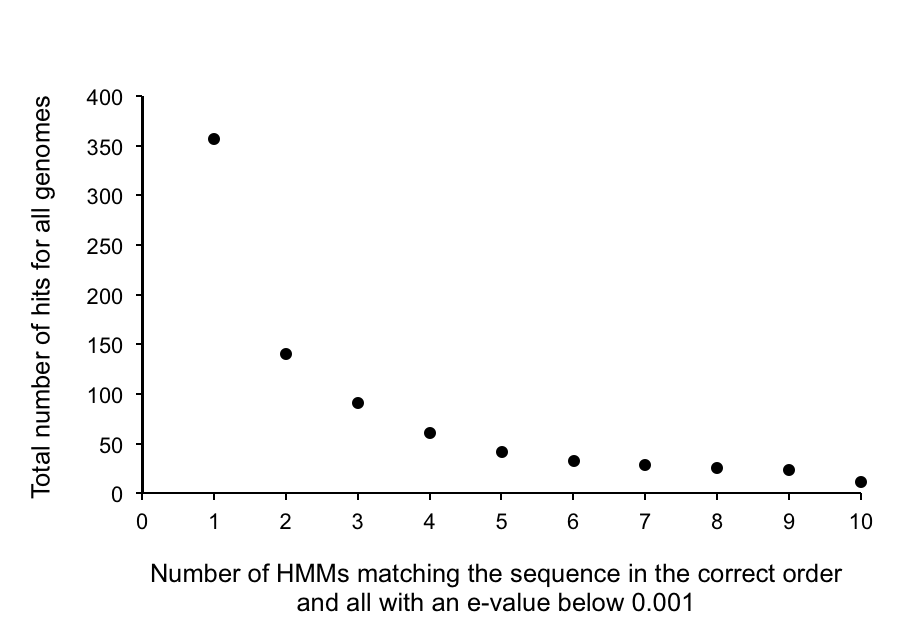


**Additional file 8.** Arthropod HMM results. Number of hits for the *Dscam-like* HMMs built from *A. mellifera, D. melanogaster*, *D. mojavensis* and *T. castaneum* and run against the translated genomes of *A. gambiae*, *A. mellifera*, *A. pisum,* *B. mori, D. pulex*, *I. scapularis,* *P. humanus humanus* and *T. castaneum*. The cut-off value is shown as a dashed line.
