# Supplementary material for: The evolution of Dscam genes across the arthropods
Source: BMC Evol Biol. 2012 Apr 13;12:53. doi: 10.1186/1471-2148-12-53 (PMC3364881; doi:10.1186/1471-2148-12-53)
Supplement: Additional file 13 — E-value distribution among putative hypervariable exons found across all arthropod species using our HMMs. The vertical dashed line marks the cut-off e-value of 0.0001. [file 1471-2148-12-53-S13.DOC]

**
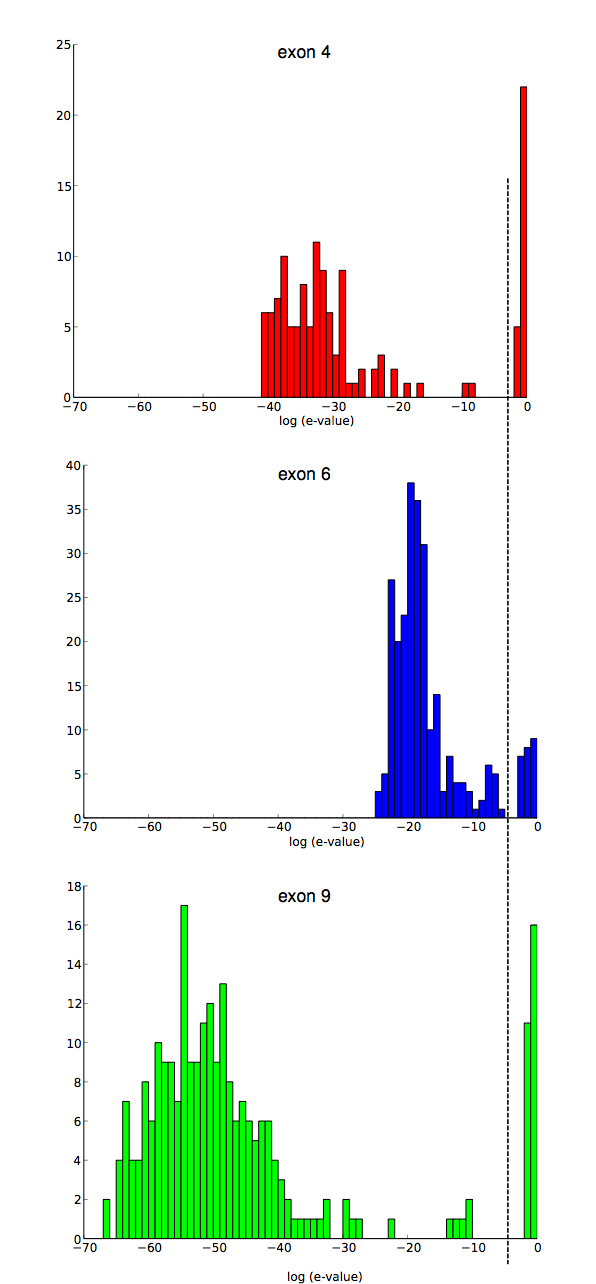
**

Occurrences

Ig2

Ig3

Ig7

**Additional file 13. E-value distribution among putative hypervariable exons found across all arthropod species using our HMMs.** The vertical dashed line marks the cut-off e-value of 0.0001
