# Supplementary material for: The evolution of Dscam genes across the arthropods
Source: BMC Evol Biol. 2012 Apr 13;12:53. doi: 10.1186/1471-2148-12-53 (PMC3364881; doi:10.1186/1471-2148-12-53)
Supplement: Additional file 15 — Tests of alternative tree topologies. The "best" tree (top; Additional files 22 &23) was tested against alternative hypotheses for the relationships between different Dscam clades by constructing alternative topologies (bottom two trees) and performing the Shimodaira-Hasegawa test. Neither of the two alternative topologies was significantly worse than the "best" tree at the 1% level. [file 1471-2148-12-53-S15.DOC]

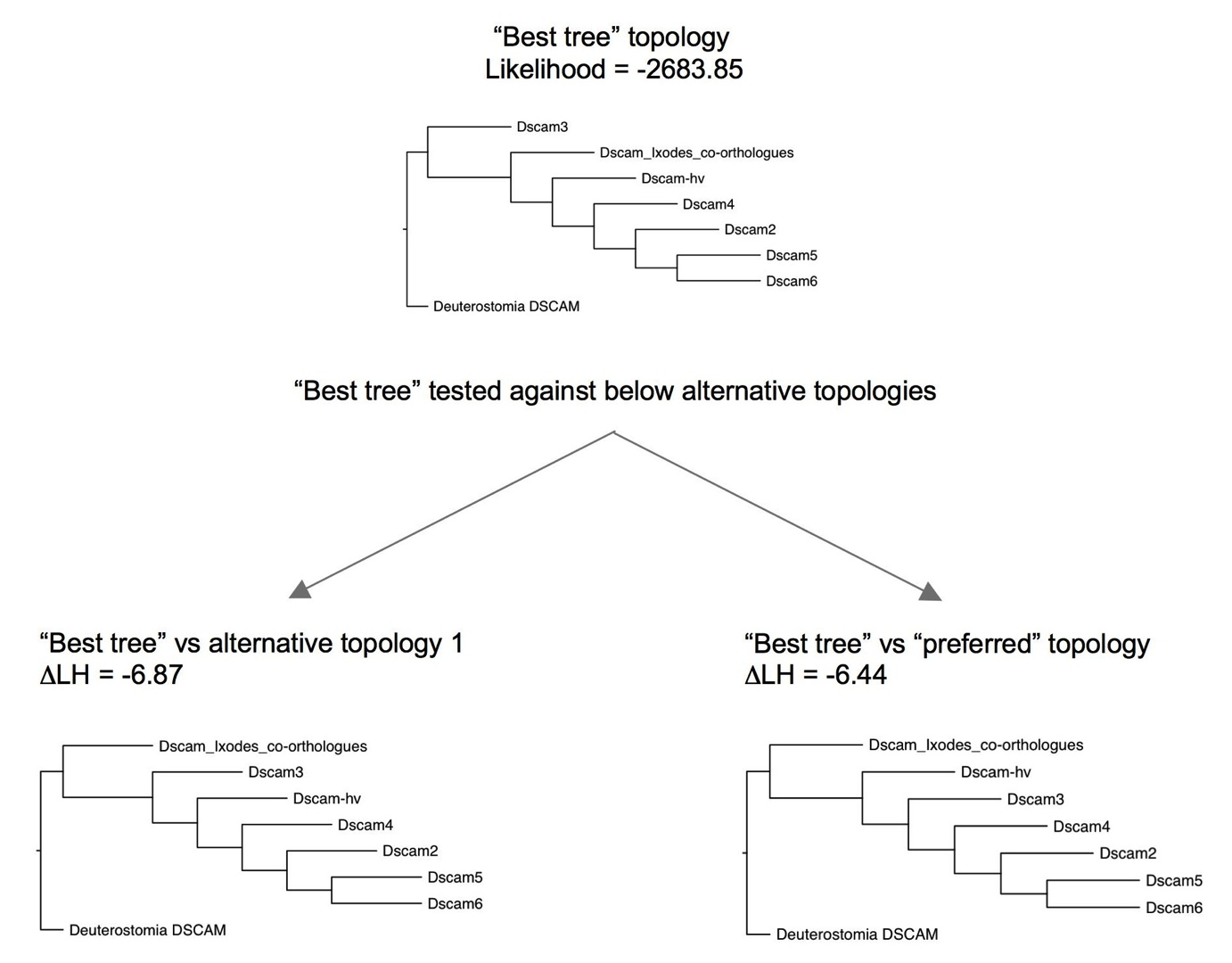


**Additional file 15. Tests of alternative tree topologies.** The “best” tree (top; Additional files 22 & 23) was tested against alternative hypotheses for the relationships between different *Dscam* clades by constructing alternative topologies (bottom two trees) and performing the Shimodaira-Hasegawa test. Neither of the two alternative topologies was significantly worse than the “best” tree at the 1% level.
