# Supplementary material for: The evolution of Dscam genes across the arthropods
Source: BMC Evol Biol. 2012 Apr 13;12:53. doi: 10.1186/1471-2148-12-53 (PMC3364881; doi:10.1186/1471-2148-12-53)
Supplement: Additional file 22 — Maximum likelihood (RAxML) phylogeny of the Dscam/DSCAM gene family, resulting in the best tree (Additional files 15 and 23). Bootstrap values (out of 100) are shown at the nodes. The vertical bars to the right are the same as in Figures 3 and 4 and follow the taxa colour codes in Figure 2. The scale bar represents 0.2 substitutions per site. [file 1471-2148-12-53-S22.DOC]

**Additional file 22. Maximum likelihood (RAxML) phylogeny of the *Dscam*/DSCAM gene family, resulting in the best tree (Additional files 15 & 23).** Bootstrap values (out of 100) are shown at the nodes. The vertical bars to the right are the same as in figs. 3 and 4 and follow the taxa colour codes in fig. 2. The scale bar represents 0.2 substitutions per site.
