# Supplementary material for: The evolution of Dscam genes across the arthropods
Source: BMC Evol Biol. 2012 Apr 13;12:53. doi: 10.1186/1471-2148-12-53 (PMC3364881; doi:10.1186/1471-2148-12-53)
Supplement: Additional file 23 — Bayesian (PhyloBayes) phylogeny of the Dscam/DSCAM gene family, resulting in the best tree (Additional file 15 & Additional file 22). Posterior probabilities are shown at the nodes. The vertical bars to the right are the same as in Figures 3 and 4 and follow the taxa colour codes in Figure 2. The scale bar represents 0.4 substitutions per site. [file 1471-2148-12-53-S23.DOC]

**Additional file 23. Bayesian (PhyloBayes) phylogeny of the *Dscam*/DSCAM gene family, resulting in the best tree (Additional files 15 & 22).** Posterior probabilities are shown at the nodes. The vertical bars to the right are the same as in figs. 3 and 4 and follow the taxa colour codes in fig. 2. The scale bar represents 0.4 substitutions per site.
