# Supplementary material for: The evolution of Dscam genes across the arthropods
Source: BMC Evol Biol. 2012 Apr 13;12:53. doi: 10.1186/1471-2148-12-53 (PMC3364881; doi:10.1186/1471-2148-12-53)
Supplement: Additional file 24 — Bayesian (PhyloBayes, relaxed clock) dated phylogeny of the Dscam/DSCAM gene family. 95% confidence intervals for divergence times (millions of years) are shown next to the key nodes. The x-axis shows the time scale in millions of years. The topology follows that of the original best tree (see Additional file 15, 22 and Additional file 23). Nodes used for fossil calibrations are shown with a grey circle, for details see materials and methods. The vertical bars follow the bar colours of taxa written in black in Figure 2. [file 1471-2148-12-53-S24.DOC]

**Additional File 24**

**Additional file 24. Bayesian (PhyloBayes, relaxed clock) dated phylogeny of the *Dscam*/DSCAM gene family.** 95% confidence intervals for divergence times (millions of years) are shown next to the key nodes. The x-axis shows the time scale in millions of years. The topology follows that of the original best tree (see Additional files 15, 22 & 23). Nodes used for fossil calibrations are shown with a grey circle, for details see materials and methods. The vertical bars follow the bar colours of taxa written in black in Figure 2.
