# Supplementary material for: The evolution of Dscam genes across the arthropods
Source: BMC Evol Biol. 2012 Apr 13;12:53. doi: 10.1186/1471-2148-12-53 (PMC3364881; doi:10.1186/1471-2148-12-53)
Supplement: Additional file 25 — Bayesian (PhyloBayes) phylogeny of all hypervariable Ig2 variants across the arthropods. A putative Ixodes scapularis Ig2 sequence is the outgroup. Bootstrap values are shown at the nodes. The scale bar represents 0.4 substitutions per site. [file 1471-2148-12-53-S25.DOC]

**Additional File 25**

**Additional file 25. Bayesian (PhyloBayes) phylogeny of all hypervariable Ig2 variants across the arthropods.** An *Ixodes scapularis* Ig2 co-ortholog is the outgroup. Bootstrap values are shown at the nodes. The scale bar represents 0.4 substitutions per site.
