# Supplementary material for: The evolution of Dscam genes across the arthropods
Source: BMC Evol Biol. 2012 Apr 13;12:53. doi: 10.1186/1471-2148-12-53 (PMC3364881; doi:10.1186/1471-2148-12-53)
Supplement: Additional file 29 — Maxiumum likelihood (RAxML) phylogeny of all hypervariable Ig3 variants across the arthropods. A putative Ixodes scapularis Ig3 sequence is the outgroup. Bootstrap values are shown at the nodes. The scale bar represents 0.4 substitutions per site. [file 1471-2148-12-53-S29.DOC]

**Additional File 29**

**Additional file 29. Maxiumum likelihood (RAxML) phylogeny of all hypervariable Ig3 variants across the arthropods.** An *Ixodes scapularis* Ig3 co-ortholog is the outgroup. Bootstrap values are shown at the nodes. The scale bar represents 0.4 substitutions per site.
