# Supplementary material for: The evolution of Dscam genes across the arthropods
Source: BMC Evol Biol. 2012 Apr 13;12:53. doi: 10.1186/1471-2148-12-53 (PMC3364881; doi:10.1186/1471-2148-12-53)
Supplement: Additional file 32 — Bayesian (PhyloBayes) phylogeny of hypervariable Ig 3 variants (exon 6) from Drosophila melanogaster and D. mojavensis. A putative Ixodes scapularis Ig3 sequence is the outgroup. Bootstrap values are shown at the nodes. The scale bar represents 1 substitution per site. [file 1471-2148-12-53-S32.DOC]

**Additional file 32. Bayesian (PhyloBayes) phylogeny of hypervariable Ig3 variants (exon 6) from *Drosophila melanogaster* and *D. mojavensis.*** A putative *Ixodes scapularis* Ig3 sequence is the outgroup. Bootstrap values are shown at the nodes. The scale bar represents 1 substitution per site.
