# Supplementary material for: The evolution of Dscam genes across the arthropods
Source: BMC Evol Biol. 2012 Apr 13;12:53. doi: 10.1186/1471-2148-12-53 (PMC3364881; doi:10.1186/1471-2148-12-53)
Supplement: Additional file 34 — Bayesian (PhyloBayes) phylogeny of hypervariable Ig7 variants (exon 9) from Drosophila melanogaster and D. mojavensis. A putative Ixodes scapularis Ig7 sequence is the outgroup. Bootstrap values are shown at the nodes. The scale bar represents 2 substitutions per site. [file 1471-2148-12-53-S34.DOC]

**Additional File 34**

**Additional file 34. Bayesian (PhyloBayes) phylogeny of hypervariable Ig7 variants (exon 9) from *Drosophila melanogaster* and *D. mojavensis.*** An *Ixodes scapularis* Ig2 co-ortholog is the outgroup. Bootstrap values are shown at the nodes. The scale bar represents 2 substitutions per site.
